# Wild-Type p53-Induced Phosphatase 1 Plays a Positive Role in Hematopoiesis in the Mouse Embryonic Head

**DOI:** 10.3389/fcell.2021.732527

**Published:** 2021-09-17

**Authors:** Wenyan He, Ying Zhang, Zhan Cao, Zehua Ye, Xun Lu, Junwan Fan, Wei Peng, Zhuan Li

**Affiliations:** ^1^China National Clinical Research Center for Neurological Diseases, Beijing Tiantan Hospital, Capital Medical University, Beijing, China; ^2^Department of Developmental Biology, School of Basic Medical Sciences, Southern Medical University, Guangzhou, China; ^3^Department of Stomatology, The First Affiliated Hospital, Sun Yat-sen University, Guangzhou, China

**Keywords:** embryonic head, hematopoietic stem cell, Wip1, microglia, pro-inflammatory factor

## Abstract

The first adult repopulating hematopoietic stem cells (HSCs) are found in the aorta-gonad-mesonephros (AGM) region, which are produced from hemogenic endothelial cells. Embryonic head is the other site for HSC development. Wild-type p53-induced phosphatase 1 (Wip1) is a type-2Cδ family serine/threonine phosphatase involved in various cellular processes such as lymphoid development and differentiation of adult HSCs. Most recently, we have shown that Wip1 modulates the pre-HSC maturation in the AGM region. However, it is not clear whether Wip1 regulates hematopoiesis in the embryonic head. Here we reported that disruption of Wip1 resulted in a decrease of hematopoietic progenitor cell number in the embryonic head. *In vivo* transplantation assays showed a reduction of HSC function after Wip1 ablation. We established that Wip1 deletion reduced the frequency and cell number of microglia in the embryonic head. Further observations revealed that Wip1 absence enhanced the gene expression of microglia-derived pro-inflammatory factors. Thus, it is likely that Wip1 functions as a positive regulator in HSC development by regulating the function of microglia in the embryonic head.

## Introduction

Hematopoietic stem cells (HSCs) provide hematopoietic progenitor cells (HPCs) and mature blood cells depending on the capacity of self-renewing and differentiation. The aorta-gonad-mesonephros (AGM) region is the site for the generation of the first HSC with long-term repopulating potential in the embryo (Muller et al., 1994). It is well known that HSCs are derived from hemogenic endothelial cells (ECs), which are produced by early arterial EC precursors (Chen et al., 2009; Kim et al., 2010; Hou et al., 2020; Howell and Speck, 2020). Previous studies have identified the regulatory molecules of AGM HSC productions, such as pro-inflammatory factors, adrenomedullin (ADM)/receptor activity-modifying protein 2 (RAMP2), and G protein-coupled receptor 56 (Gpr56) (Li et al., 2014, 2019; Solaimani Kartalaei et al., 2015; Dzierzak and Bigas, 2018; Mariani et al., 2019; Yvernogeau et al., 2020). Moreover, definitive erythro-myeloid progenitors (EMPs) are emerged from ECs in the yolk sac beginning at embryonic day (E) 8/8.5. EMPs are phenotypically defined by a cocktail of markers CD41, cKit, and CD16/32, positively distinguishing EMPs from embryonic HPCs in the yolk sac (McGrath et al., 2015; Frame et al., 2016). Recent fate-mapping studies have evidently demonstrated that EMPs from yolk sac contribute macrophages in the embryonic head (microglia) during conditions of hemostasis (Gomez Perdiguero et al., 2015).

Embryonic head is the other site for hematopoietic stem and progenitor cell (HSPC) emergence from our previous study. Functional transplantation and lineage tracing data have demonstrated that HSPCs are produced from the vascular of head (Li et al., 2012). These HSPCs in the embryonic head display a single cell phenotype, not forming “hematopoietic clusters,” which appeared in the AGM region (Iizuka et al., 2016; Li et al., 2016), suggesting differences in the regulation of head hematopoiesis. Recently, we found that head CD45^+^F4/80^+^CD11b^+^ macrophages (microglia) acted as microenvironmental cellular regulators, promoting the process of endothelial to hematopoietic transition in the embryonic head by secreting the pro-inflammatory factor tumor necrosis factor-α (*TNF-*α) (Li et al., 2019). However, the regulatory mechanisms of hematopoiesis in the embryonic head remain to be investigated.

Wild-type p53-induced phosphatase 1 (Wip1) is encoded by protein phosphatase magnesium-dependent 1 delta (*PPM1D*), which is a critical regulator involved in various cellular processes (Uyanik et al., 2017), including neurogenesis (Zhu et al., 2009), tumorigenesis (Belova et al., 2005; Demidov et al., 2007), cell aging, neutrophil maturation (Liu et al., 2013; Sun et al., 2014), and lymphoid development (Yi et al., 2015). Specially, Chen et al. (2015) showed that Wip1 affects the function of HSCs *via* p53 and mammalian target of rapamycin complex 1 (mTORC1) pathways. Wip1-deficient embryos were viable; however, the defects in growth, organ structure, and fertility were observed in postnatal mice (Choi et al., 2002). Most recently, we reported that Wip1 affects the pre-HSC maturation and HPC development by altering cell cycle in the embryonic AGM region (He et al., 2021). However, the effects of Wip1 on mediating embryonic head hematopoiesis have yet to be established.

In this study, we find that Wip1 is required for the development of definitive HSPCs in the embryonic head. Moreover, Wip1 affects the development/function of microglia by enhancing pro-inflammatory factor gene expression. Our findings suggest that Wip1 regulates hematopoiesis in the embryonic head region by altering the pro-inflammatory factor status.

## Results

### Wild-Type p53-Induced Phosphatase 1 Deficiency Results in the Reduction of Hematopoietic Progenitor Cells in the Embryonic Head

**FIGURE 1:**
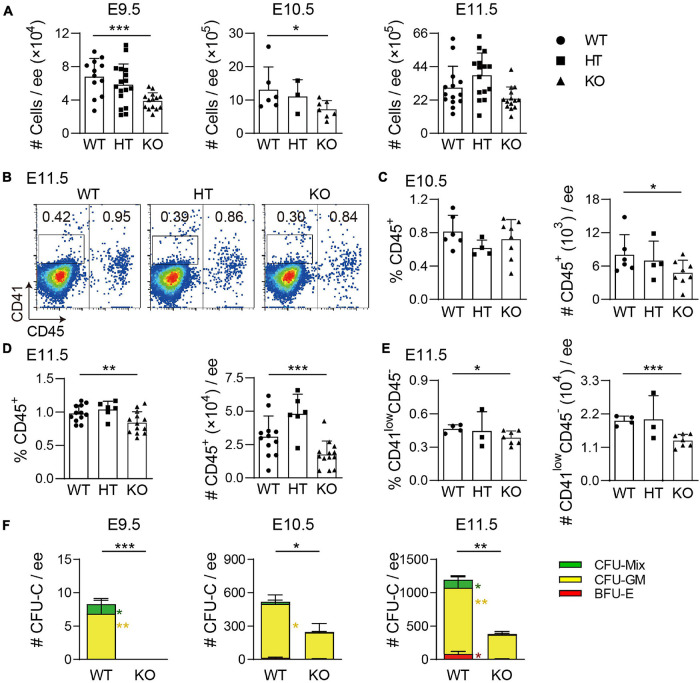
Wip1 deficiency results in the reduction of hematopoietic progenitor cell number in the embryonic head. **(A)** The reduction of the total numbers in the E9.5–E11.5 embryonic head. E9.5, *n* = 5; E10.5, *n* = 3; E11.5, *n* = 8; *p < 0.05, ***p < 0.001. **(B)** Representative flow cytometry analysis of CD41^*low*^CD45^–^ and CD45^+^ cells in the E11.5 embryonic head. **(C,D)** The percentages and absolute numbers of CD45^+^ cells in the E10.5–E11.5 embryonic head. E10.5, *n* = 3; E11.5, *n* = 6; *p < 0.05, **p < 0.01, ***p < 0.001. **(E)** The percentage and absolute number of CD41^*low*^CD45^–^ cells in the E11.5 embryonic head. *n* = 3; *p < 0.05, ***p < 0.001. **(F)** Colony-forming unit-culture (CFU-C) assays showed the number of CFU-Cs in the E9.5–E11.5 embryonic head. Numbers of colony type are indicated by bar color. E9.5, *n* = 3; E10.5, *n* = 2; E11.5, *n* = 4; *p < 0.05, **p < 0.01, ***p < 0.001.

Recently, we have reported that Wip1 is involved in the regulation of HSPC development in the AGM region. To test whether Wip1 plays a role in hematopoiesis of the embryonic head, *Wip1* homozygous deficient embryos (*Wip1^–/–^*, KO) were obtained by crossing *Wip1* heterozygous deficient (*Wip1*^+/–^, HT) mice. A reduced head size was observed under microscope from E9.5 to E12.5 (data not shown). But the total cell numbers were decreased only in E9.5 and E10.5 Wip1-deficient head compared with wild-type (WT) controls and not in E11.5 (Figure 1A). However, the head cell viabilities in all stages we detected by flow cytometry were comparable (Supplementary Figure 1A). Flow cytometry analysis showed that the percentage of CD45^+^ cells was significantly increased at E9.5 (0.23 ± 0.05% vs. 0.09 ± 0.03%) but decreased at E11.5 Wip1*^–^*^/^*^–^* head (0.84 ± 0.05% vs. 0.98 ± 0.04%); however, it was not changed in E10.5 head (0.70 ± 0.08% vs. 0.80 ± 0.08%) (Figures 1B–D and Supplementary Figure 1B). The absolute numbers of CD45^+^ cells were reduced dramatically in the E10.5 (4.78 ± 0.80 × 10^3^ vs. 8.03 ± 1.46 × 10^3^) and E11.5 (1.76 ± 0.28 × 10^4^ vs. 3.10 ± 0.45 × 10^4^) Wip1*^–^*^/^*^–^* head but not in E9.5 (Figures 1C,D and Supplementary Figure 1C). Moreover, Wip1 deletion decreased the percentage (0.39 ± 0.02% vs. 0.47 ± 0.02%) and the cell number (33% reduction, 1.32 ± 0.08 × 10^4^ vs. 1.98 ± 0.08 × 10^4^) of CD41^*low*^CD45^–^ cells (including HPCs) in the E11.5 head (Figure 1E), indicating impaired hematopoietic development in the embryonic head. Furthermore, to test the hematopoietic progenitor function, colony-forming unit-culture (CFU-C) assays confirmed a dramatic decrease in HPC function from E9.5 to E11.5 Wip1*^–^*^/^*^–^* head compared to WT head, including the reductions of burst forming unit-erythroid (BFU-E) of E11.5 (5 ± 5 vs. 80 ± 42), CFU-granulocyte-macrophage (CFU-GM) in E9.5–E11.5 (E9.5, 0 ± 0 vs. 7 ± 2; E10.5, 238 ± 81 vs. 486 ± 80; E11.5, 365 ± 50 vs. 993 ± 165), and CFU-granulocyte-erythroid-macrophage-megakaryocyte [CFU-GEMM(Mix)] in E9.5 (0 ± 0 vs. 1 ± 1) and E11.5 (10 ± 10 vs. 120 ± 61) (Figure 1F), which is similar to the trend of CD41-enriched HPCs in the embryonic head (Figure 1E) and AGM region (He et al., 2021). Meanwhile, the reduced morphologic size of CFU-Cs was seen in all stages, and the CFU-Cs per input cell number were decreased dramatically in E9.5 and E11.5 (Supplementary Figures 1D,E). These results suggest that Wip1 is involved in HPC development of the embryonic head.

### Loss of Wild-Type p53-Induced Phosphatase 1 Results in Impaired Hematopoietic Stem Cell Function in the Mid-Gestation Head

**FIGURE 2:**
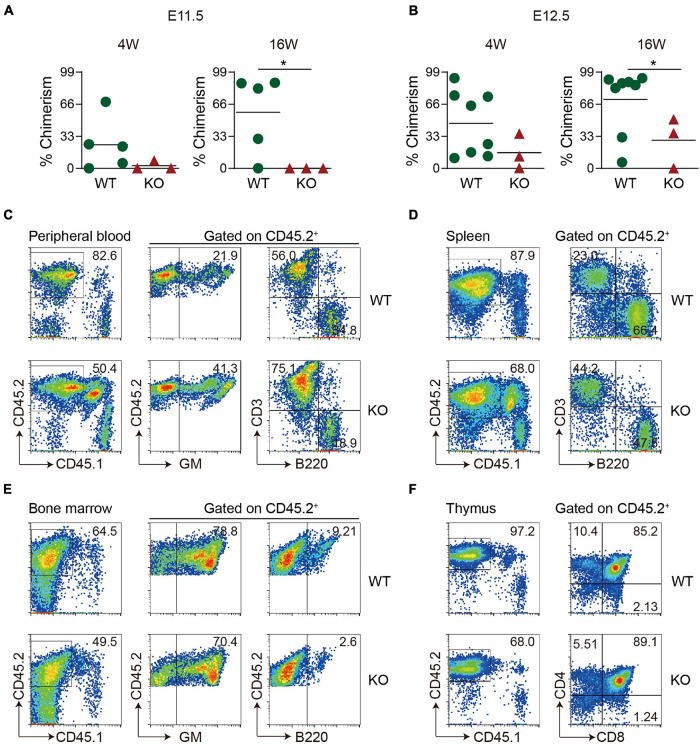
Hematopoietic stem cell activity is decreased in Wip1 knockout head of the E11.5–E12.5 embryo. **(A,B)** Direct transplantation assay showing the repopulating ability of E11.5 and E12.5 wild-type (WT) and *Wip1*^–/–^ embryonic head. Symbols represent the donor chimerism of CD45.2^+^ cells in peripheral blood of individual recipients in 4 weeks (left) and 16 weeks (right) posttransplantation. *p < 0.05. **(C–F)** Representative analysis of multilineage output of repopulated recipients receiving E12.5 head cells. The donor contribution is revealed by the presence of the donor of CD45.2^+^ cells in the myeloid (Gr-1/Mac1), B lymphoid (B220), and T lymphoid (CD3, CD4, and CD8) cells of the peripheral blood **(C)**, spleen **(D)**, bone marrow **(E)**, and thymus **(F)** in two representative recipients at 6 months posttransplantation. The recipients were injected with 1 embryo equivalent (ee) of E11.5 and E12.5 head cells.

To see whether Wip1 deletion affects HSC function, *in vivo* transplantation assays were performed. E11.5–E12.5 head cells were injected intravenously into irradiated adult recipients, and the chimerism was detected in the peripheral blood of recipients at 4 and 16 weeks posttransplantation. None of recipients received E11.5 Wip1*^–^*^/^*^–^* head cells were repopulated at 4 and 16 weeks, although four out of five recipients (chimerism 57.6 ± 18.0%) by injecting with WT head showed long-term, high-level, multilineage repopulation at E11.5 (Figure 2A). Unexpectedly, the repopulated ratios from the E12.5 Wip1*^–^*^/^*^–^* head cell with lower chimerism (28.9 ± 15.0% vs. 70.9 ± 11.6%) were reduced significantly at 16 weeks posttransplantation compared to those in the control group (2/3 vs. 7/8) (Figure 2B). The profile of multilineage output was similar to our previous data, with an increased trend of myeloid and T lymphoid lineage output, at the expense of B lymphoid output in the peripheral blood of Wip1*^–^*^/^*^–^* head-derived recipients (Figure 2C). Moreover, the Wip1^–/–^ head-derived HSC attributed to hematopoietic cells in the various hematopoietic organs (spleen, bone marrow, and thymus) (Figures 2D–F) demonstrated the capacity for multilineage engraftment. In conclusion, these findings indicate that Wip1 affects HSC activity definitively in the E11.5–E12.5 head.

### Deletion of Wild-Type p53-Induced Phosphatase 1 Affects Macrophage Development in the Embryonic Head but Not in the Yolk Sac

**FIGURE 3:**
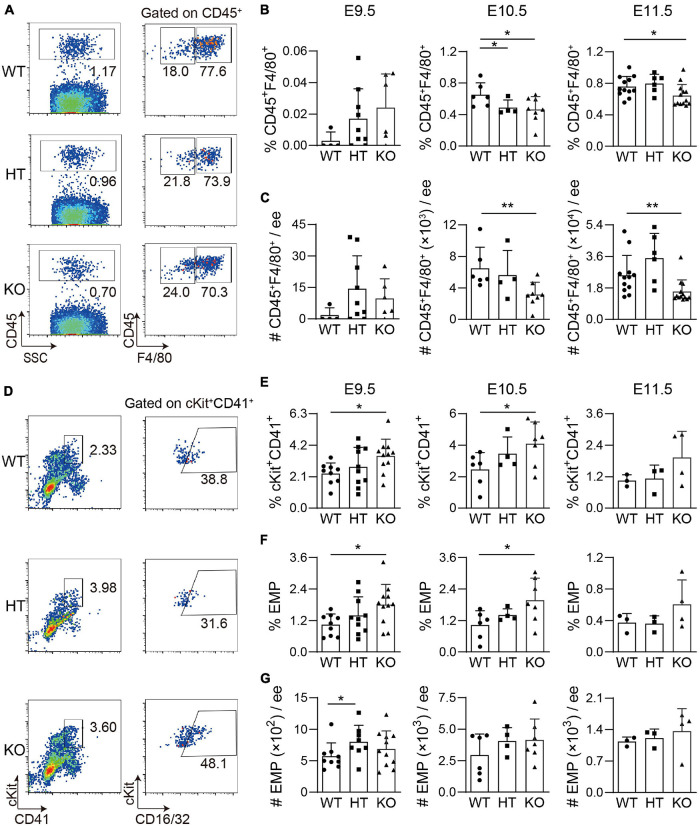
The development of microglia and related cell population in the Wip1 ablation embryonic head/yolk sac. **(A)** Representative flow cytometry analysis of CD45^+^ cells and F4/80^+^ microglia of the E11.5 embryonic head. **(B)** The percentages of CD45^+^F4/80^+^ E9.5–E11.5 embryonic head. *p < 0.05. **(C)** The absolute numbers of F480^+^CD45^+^ cells in E9.5–E11.5 embryonic head. E9.5, *n* = 4; E10.5, *n* = 3; E11.5, *n* = 6; **p < 0.01. **(D)** Representative flow cytometry analysis of ckit^+^CD41^+^ cells and erythro-myeloid progenitors (EMPs) in the E9.5 yolk sac. **(E)** The percentages of ckit^+^CD41^+^ cells in the E9.5-E11.5 yolk sac. E9.5, *n* = 5; E10.5, *n* = 3; E11.5, *n* = 3; *p < 0.05. **(F,G)** The percentages and absolute numbers of EMPs (ckit^+^CD41^+^CD16/32^+^) in the E9.5–E11.5 yolk sac. E9.5, *n* = 5; E10.5, *n* = 3; E11.5, *n* = 3; *p < 0.05.

Our recent studies have shown that head microglia cells are pivotal as positive hematopoietic regulators. As we mentioned, CD45^+^ cells were decreased after Wip1 deletion. To uncover whether Wip1 regulates the number and function of embryonic head microglial cells, F4/80 as a microglia marker was used for flow cytometry analysis. Compared to WT head, the deletion of Wip1 resulted in a reduction of microglia proportions (CD45^+^F4/80^+^%) in E10.5 (0.46 ± 0.06% vs. 0.65 ± 0.06%) and E11.5 (0.65 ± 0.04% vs. 0.76 ± 0.04%) but not in E9.5 (0.024 ± 0.009% vs. 0.003 ± 0.003%). In contrast, the percentages of CD45^+^ F4/80^–^ were increased in E10.5 (Figures 3A,B and Supplementary Figure 2A), in line with the impaired hematopoietic activity. Similarly, the cell number of microglia was radically reduced in E10.5 (51% reduction, 3.16 ± 0.55 × 10^3^ vs. 6.50 ± 1.09 × 10^3^) to E11.5 (37% reduction, 1.59 ± 0.19 × 10^4^ vs. 2.52 ± 0.33 × 10^4^) but not in E9.5. Furthermore, the numbers of CD45^+^ F4/80^–^ were comparable (Figure 3C and Supplementary Figure 2B). The above results indicated that Wip1 loss reduced the microglia cell number in the embryonic head, suggesting impaired physiologic hematopoietic support.

In contrast, the percentages and absolute numbers of macrophage (CD45^+^F480^+^CD11b^+^) were comparable in the E11.5 Wip1^–/–^ yolk sac, as well as the same trend of CD45^+^ cells (Supplementary Figures 3A–D). As is known, microglia are derived from the EMPs of the yolk sac (Gomez Perdiguero et al., 2015), so we used flow cytometry analysis to define the effects on EMPs in the yolk sac of Wip1 deletion by using the cocktail of cKit, CD41, and CD16/32. We found that Wip1 ablation increased the percentages of cKit^+^CD41^+^ cells (including HPCs) in the E9.5 (3.49 ± 0.33% vs. 2.31 ± 0.24%) and E10.5 (4.10 ± 0.52% vs. 2.47 ± 0.44%) but not in the E11.5 yolk sac (1.94 ± 0.49% vs. 1.05 ± 0.13%) (Figures 3D,E). Furthermore, the percentage of EMP (cKit^+^CD41^+^CD16/32^+^) was enhanced at the same stage of yolk sac (E9.5: 1.80 ± 0.23% vs. 1.04 ± 0.14%, E10.5: 1.97 ± 0.32% vs. 1.03 ± 0.22%). However, the numbers of c-Kit^+^CD41^+^ cells and EMPs were unchanged in the E9.5–E11.5 Wip1*^–^*^/^*^–^* yolk sac compared to WT (Figures 3F,G and Supplementary Figure 3E), possibly due to the reduction of total number. These data indicate that Wip1 is indeed involved in the EMP development of the yolk sac that might affect the migration of EMP in the yolk sac to the head, leading to the reduction of microglia.

### Deletion of Wild-Type p53-Induced Phosphatase 1 Affects Pro-inflammatory Factor Expression in Microglia

**FIGURE 4:**
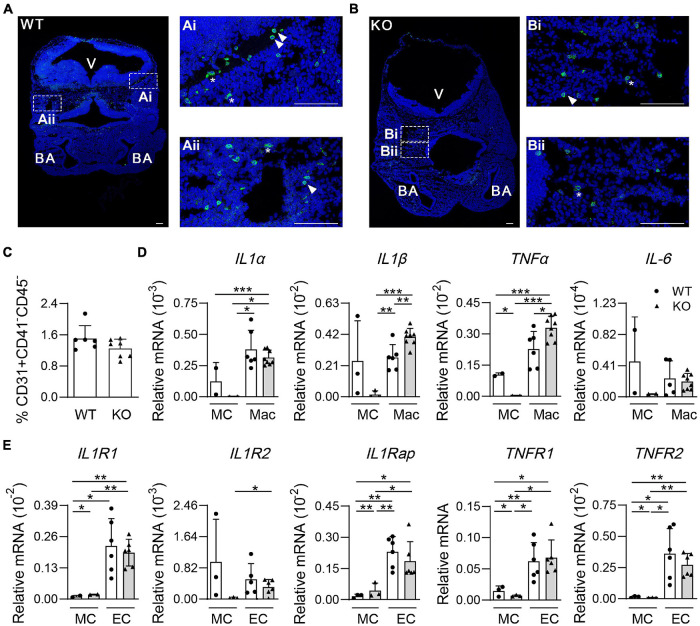
The morphology of microglia and gene expression of pro-inflammatory pathways in the mouse embryonic head. **(A,B)** Representative confocal images of immunostaining by F4/80 (microglia) and Hoechst 33342 in the E11.5 head sections. Wild-type (WT) **(A)** 48 sp., knockout (KO) **(B)** 45 sp. Arrowhead indicated round microglia. Star indicated non-round microglia. V, ventricle; BA, branchial arches. Bar = 100 μm. **(C)** The percentages of endothelial cells (CD31^+^CD41^+^CD45^–^, ECs) in the E11.5 embryonic head. *n* = 4. **(D)** Relative mRNA expression of *IL-1*α, *IL-1*β, *TNF-*α, and *IL-6* normalized to β-actin in the microglia (CD45^+^F4/80^+^CD11b^+^, Mac) and mesenchymal cells (CD31^–^CD45^–^CD41^–^, MCs) of the E11.5 head by qRT-PCR. *n* ≥ 2; *p < 0.05, **p < 0.01, ***p < 0.001. **(E)** Relative mRNA expressions of *IL-1* and *TNF* receptor (*IL1R1*, *IL1R2*, *IL1Rap*, *TNFR1*, and *TNFR2*) in ECs (CD31^+^CD41^–^CD45^–^) and mesenchymal cells (CD31^–^CD45^–^CD41^–^, MCs) of the E11.5 head were determined by qRT-PCR. *n* ≥ 2. *p < 0.05, **p < 0.01, ***p < 0.001.

To observe the morphology of microglia, immunostaining assays of cryosections were performed. There were fewer F4/80^+^ cells in Wip1^–/–^ head sections, including round and non-round microglia cells, in line with flow cytometry analysis data (Figures 3B,C). Wip1 deletion appeared to change the morphology of microglia, which might be related to the immune function (Figures 4A,B). It is known that microglial cells positively regulate the hemogenic potential of ECs through pro-inflammatory factors in the embryo (Li et al., 2019). qRT-PCR was performed to check the expression of pro-inflammatory factors, such as interleukin-1α (*IL-1*α), *IL-1*β, *TNF*-α, and *IL-6*, of microglia (CD45^+^F4/80^+^CD11b^+^, Mac)/mesenchymal cells (CD31*^–^*CD45*^–^*CD41*^–^*, MCs) and their receptors of ECs (CD31^+^CD41^–^CD45^–^, EC)/MC. The percentages of EC were comparable after Wip1 deficiency (Figure 4C). We found that the expression of pro-inflammatory factors (*IL-1*α, *IL-1*β, and *TNF*-α) appeared to be higher in the microglia of head compared to MC in the Wip^–/–^ head, which is similar to the expression of most cognate receptors (Figures 4D,E). The expressions of *IL-1*β and *TNF*-α were significantly increased by >30% in Wip1^–/–^ head compared with those in control (Figure 4D), although the mRNA levels of *IL-1*α and *IL-6* were unaltered in the Wip1^–/–^ microglial cells. Interestingly, the receptors relevant to the *IL-1* and *TNF*-α pathways were not changed in the endothelial when Wip1 was ablated (Figure 4E). Unexpectedly, the expressions of pro-inflammatory factors (*IL-1*α, *IL-1*β, and *TNF*-α) were decreased in the macrophage of Wip1^–/–^ yolk sac compared to WT but not in the MC fractions. The cognate receptors such as *IL1R2*, *IL1Rap*, and *TNFR2* were expressed less in the EC fraction; however, the expressions of *TNF1R1* and *TNFR2* were increased in the MC fractions of yolk sac (Supplementary Figures 4A,B). These different regulating effects of Wip1 on pro-inflammatory factor pathways between the head and the yolk sac indicate the specific regulatory mechanisms in distinct hematopoietic tissues. These results imply that Wip1 probably influences the secretion of pro-inflammatory factors from microglia, thereby affecting hematopoietic function.

## Discussion and Conclusion

Here, we have identified a novel role for Wip1 in hematopoietic development in the embryonic head, expanding its known role in HSC development and differentiation (Liu et al., 2013; Chen et al., 2015; Yi et al., 2015). Wip1 influences the definitive HSPC function in the embryonic head, regulates the microglia numbers, and probably alters the secretion of the pro-inflammatory factor. Intriguingly, Wip1 is essential for EMPs but not for macrophage development (cell number) in the embryonic yolk sac. Although we do not precisely demonstrate Wip1 function in the specific cells because of mouse model limitation, we have found that Wip1 plays an important role in the embryonic head hematopoiesis. And, this is the first demonstration that Wip1 is a positive regulator of definitive HSPCs in the embryonic head.

### Wild-Type p53-Induced Phosphatase 1 Regulates Hematopoietic Development in the Embryonic Head

As *Wip1* is expressed in the key cell types of hematopoietic development from a previous report (Zhou et al., 2016). Chen et al. (2015) have displayed that *Wip1* is highly expressed in adult bone marrow HSCs but decreased with age, exhibiting multifaceted HSC aging phenotype and impaired HSC activity. HSCs in E11.5–E12.5 embryonic head have a lower capacity of engraftment after Wip1 deletion, with increase of T/myeloid lineage output at the expense of B lymphoid cells, consistent with previous reports that Wip1 ablation impaired B-cell differentiation. Moreover, no transplantable HSCs were found in the E11.5 head region. Noticeably, less HSCs with lower engraftment ability were observed in the E12.5 head, which may be from or delayed functional HSC or other hematopoietic tissues *via* circulation. Wip1 knockout mice showed much less numbers of HPCs and hematopoietic cells by immune phenotype (CD41^*low*^CD45^–^ and CD45^+^, respectively) and functional assay (CFU-Cs) in the head of embryo at different time points as well as that in the yolk sac, AGM region, and fetal liver, respectively (He et al., 2021), indicating the time effects of Wip1 deletion on the development of distinct hematopoietic populations. Additionally, the reduction of total cell number is not the reason for the decrease in HPCs, since the CFU-Cs per cell number input were reduced dramatically in the E9.5 and E11.5 head at least.

### Wild-Type p53-Induced Phosphatase 1 Is Efficient in Regulating Erythro-Myeloid Progenitor Formation

Erythro-myeloid progenitors identified by the specific surface markers are generated from ECs in the yolk sac, which also go through endothelial to hematopoietic cell transition (McGrath et al., 2015; Frame et al., 2016). Specific regulatory mechanisms are involved in the process compared to the AGM endothelial *trans*-differentiation. In our study, the number and percentage of macrophages were not affected by Wip1 deletion in the embryonic yolk sac, although the pro-inflammatory factor pathways were changed. Interestingly, we have found that Wip1 negatively regulated EMP proportions in the yolk sac. As expected, the absolute number of EMPs failed to be altered because of the reduction of total number, which was not in line with definitive HSPC phenotype (He et al., 2021). A possible explanation is that the expressions of hematopoietic transcription factors Runx1 and Gata2 are significantly increased in Wip1^–/–^ yolk sac. Therefore, Wip1 regulates the EMP production from yolk sac ECs in the embryo; however, the specific regulatory mechanisms need to be further investigated in the future.

### Wild-Type p53-Induced Phosphatase 1 Influences Microglial Development

Head microglia are the only resident macrophages from yolk sac during hemostasis (Gomez Perdiguero et al., 2015; Li and Barres, 2018). Except microglia, it has been shown that yolk sac-derived EMPs also migrate to the embryonic fetal liver and other tissues (Hoeffel et al., 2015). We also found that Wip1 deletion resulted in a strong decrease of CD45^+^ cells (mature hematopoietic cells and hematopoietic progenitor/stem cells) along with the dramatic reduction of microglia, contrasting to that in the AGM region and yolk sac (He et al., 2021) (data not shown), indicating that Wip1 is essential for microglial development. Along with the trend of EMP in the yolk sac, Wip1 might change the migration of EMP to influence microglia development. More details concerning their migration are worthy of further study.

Functional HSCs in the embryonic stage are educated *via* several processes including endothelial to hematopoietic cell transition and pro/pre-HSC maturation (Taoudi et al., 2008; Rybtsov et al., 2011, 2014). Wip1 indeed regulates pre-HSC maturation by cell cycle modulation in the AGM region. In the embryonic head, only single hematopoietic cell forms were attached to the vascular without classic “hematopoietic clusters” (Li et al., 2016; Iizuka et al., 2016), indicating the differences of hematopoietic niche. Pro-inflammatory factors derived from macrophages (including microglia) like *TNF*-α, interferon (IFN)-γ, *IL-1*α, and *IL-1*β made influences on hematopoietic cell formation of the aorta and head, partially dependent on the concentration (Li et al., 2014, 2019; Mariani et al., 2019). In the Wip1 knockout head, the gene expression of pro-inflammatory factors on microglia was enhanced more than 30% compared with WT control; however, the existence of microglial cells failed to rescue the hematopoietic cell production of EC *in vitro* co-culture system (data not shown). There are some possible reasons, as follows: (1) The total number of microglia was reduced by more than 36% in the Wip1^–/–^ head, which compensates for the increase of gene expression; (2) The dependence of concentration is difficult to control in the *in vitro* experiments; (3) Wip1 deletion in germ line cells, including other niche cells, resulted in a more severe phenotype *in vivo*.

In summary, our study provides an additional role for Wip1 in HSC and progenitor cell development in the embryonic head. Moreover, Wip1 indeed modulates microglia development, specially regulating the microglia-derived pro-inflammatory factors. Therefore, we have shown that Wip1 as a hematopoietic regulator, which may provide some theoretical and practical implications to support regenerative medicine.

## Materials and Methods

### Animals

WT C57BL/6-Ly5.2 and *Wip1*^+/–^ heterogeneous-Ly5.2 mice were used for timed matings, and C57BL/6-Ly5.1 mice (8–12 weeks) were used as transplantable recipients. *Wip1* mutant embryos were generated by crossing *Wip1*^+/–^ males with *Wip1*^+/–^ females. Embryos (E9.5–E12.5) were staged by counting somite pairs. Head and yolk sac were dissected, and tails were used for genotyping. Mice were housed in the animal facilities, and experimentation complied with the ethics committee of Southern Medical University.

### Antibodies

CD41 (MWReg30), CD117 (2B8), CD45 (30-F11), and CD31 (MEC13.3) antibodies were purchased from BD Pharmingen. CD16/32 (93), 7-AAD, and Hoechst 33342 were purchased from Invitrogen, and F4/80 (BM8) and CD11b (M1/70) were purchased from BioLegend.

### Hematopoietic Progenitor and Stem Cell Assays

Single-cell suspensions from head were plated into MethoCult GF M3434 with Cytokines (Stem Cell Technologies) for CFU-C assays. Hematopoietic colonies were counted after 10 days of culture and then calculated according to embryo equivalent (ee). BFU-Es, CFU-GMs, and CFU-Mixes were clarified in the total CFU-C counting. Cells from the embryonic head were injected intravenously into irradiated recipients (9.0 Gy Cobalt-60-irradiation, split dose). Peripheral blood was taken from recipients (at 4 and 16 weeks) for Ly5.1-/Ly5.2-specific flow cytometry analysis. Recipients were considered repopulated when ≥10% of cells were donor-derived.

### Flow Cytometry Assay

Cells from the embryonic head or yolk sac, cultures, and adult hematopoietic tissues were stained by fluorescence conjugated antibodies for 30 min on ice. Sorted cells were collected for co-culture or in lysis buffer for RNA extraction. 7-AAD or Hoechst 33342 staining was performed for excluding dead cells. Flow cytometry analysis or sorting was performed on CytoFlex (Beckman Coulter), MoFlo XDP (Beckman Coulter), or Aria II (BD Biosciences). FACS data were analyzed with FlowJo software.

### Immunostaining Assays

Dissected E11.5 embryos were fixed in 2% paraformaldehyde at 4°C for 30–60 minutes and embedded in OCT. And then embryos were equilibrated in 20% sucrose/phosphate buffered saline (PBS) at 4°C overnight and then embedded in Tissue Tek before freezing. Ten-micrometer cryosections were prepared. Endogenous biotin activity was blocked by Avidin/Biotin blocking kit. The fixed head sections were incubated with primary antibody (F4/80, D2S9R) or secondary antibody [Anti-Rabbit Alexa Fluor 488 IgG (H + L)] into PBS-block [PBS containing 0.05% Tween and 1% bovine serum albumin (BSA)] overnight and washed three times in PBS-T (PBS with 0.05% Tween). Samples were stained with Hoechst 33342 for 10 min at room temperature and then mounted with mounting buffer. Images were acquired with an inverted confocal microscope (Zeiss LSM 880) and processed using Zeiss Zen.

### Gene Expression Analysis

RNA from sorted cells was extracted by using TRIzol reagent (Sigma) plus Glycogen (Macklin, China), and cDNA was reversed transcribed with HiScript III RT SuperMix (+gDNA wiper) for qPCR (Vazyme). Real-time PCR was performed by using ChamQ SYBR qPCR Master Mix (Vazyme) and detected on LightCycler 96 system (Roche). Sequences of primers were used according to a previous report (Li et al., 2019).

### Statistical Analysis

All data are presented as mean ± SEM. Student’s *t*-test was used for comparison of various groups. *p* < 0.05 was considered statistically significant.

## Data Availability Statement

The raw data supporting the conclusions of this article will be made available by the authors, without undue reservation.

## Ethics Statement

The animal study was reviewed and approved by the Ethics Committee of Southern Medical University.

## Author Contributions

WH and YZ carried out the experiments, data collection, and analysis. ZC performed immunostaining. ZY helped in genotyping. XL helped in the flow cytometry analysis. WP and JF gave material support. ZL and WH contributed to the study design and prepared the manuscript. All authors contributed to the article and approved the submitted version.

## Conflict of Interest

The authors declare that the research was conducted in the absence of any commercial or financial relationships that could be construed as a potential conflict of interest.

## Publisher’s Note

All claims expressed in this article are solely those of the authors and do not necessarily represent those of their affiliated organizations, or those of the publisher, the editors and the reviewers. Any product that may be evaluated in this article, or claim that may be made by its manufacturer, is not guaranteed or endorsed by the publisher.
