# Supplementary material for: Wild-Type p53-Induced Phosphatase 1 Plays a Positive Role in Hematopoiesis in the Mouse Embryonic Head
Source: Front Cell Dev Biol. 2021 Sep 17;9:732527. doi: 10.3389/fcell.2021.732527 (PMC8484912; doi:10.3389/fcell.2021.732527)
Supplement: Supplementary file 1 [file Data_Sheet_1.pdf]

FIGURE S1.

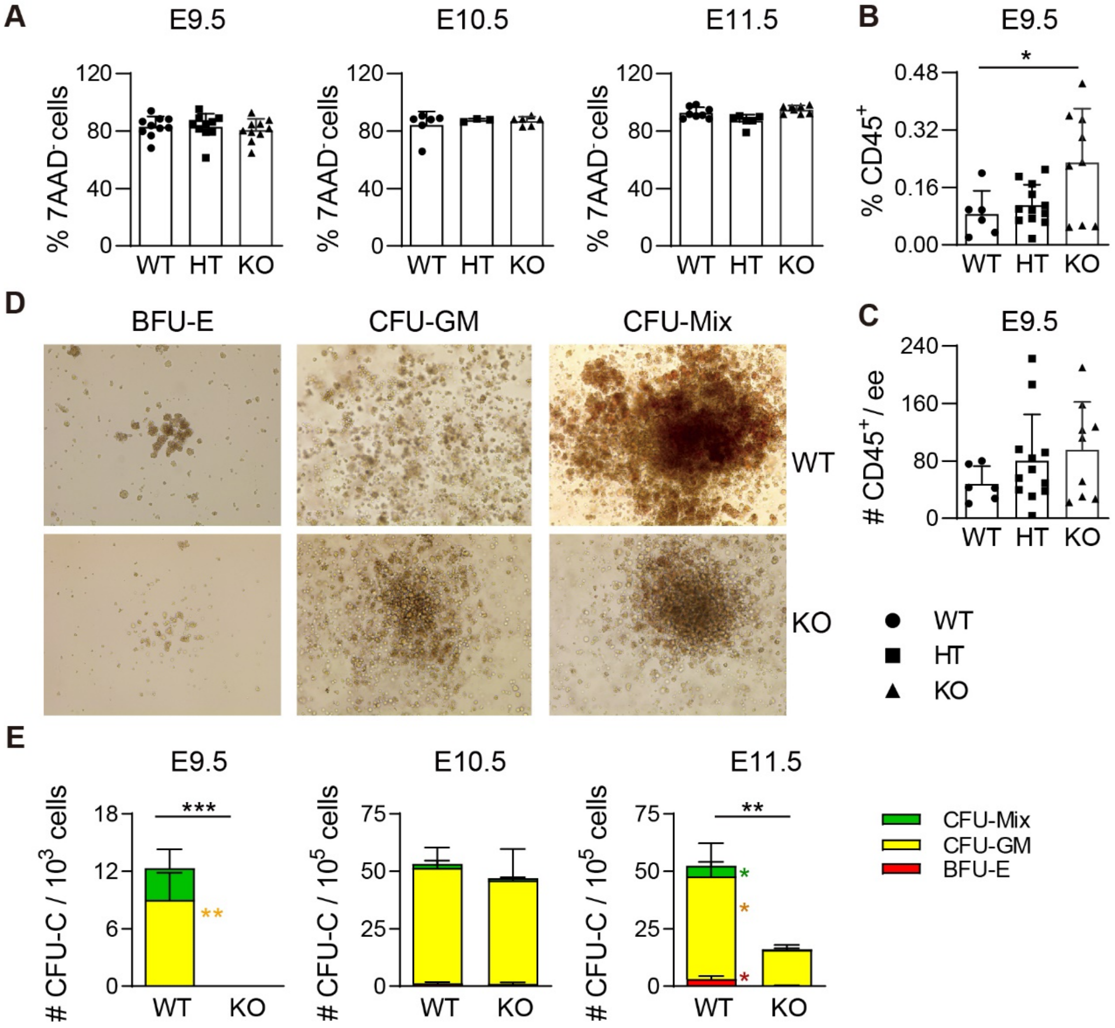

FIGURE S1 | Wip1 ablation results in the reduction of hematopoietic progenitor cell number in the embryonic head. (A) Flow cytometry analysis showing viable cells by 7AAD staining in the E9.5-E11.5 WT and $Wip1^{-/-}$ head. E9.5 n=5; E10.5 n=3; E11.5 n=5. **(B, C)** The percentages and absolute numbers of $CD45^+$ cells in E9.5 embryonic head. n=6; **P*< 0.05. **(D)** Morphology of colony forming unit-culture （CFU-C） hematopoietic colonies derived from E11.5 embryonic head cells. **(E)** CFU-C assay showing the number of colonies per $1x10^3$ or $1x10^5$ embryonic head cells from E9.5 to E11.5. Colony types are indicated by colored bars. E9.5 n=3; E10.5 n=2; E11.5 n=4; **P*< 0.05, ***P*< 0.01, ****P*< 0.001.

FIGURE S2.

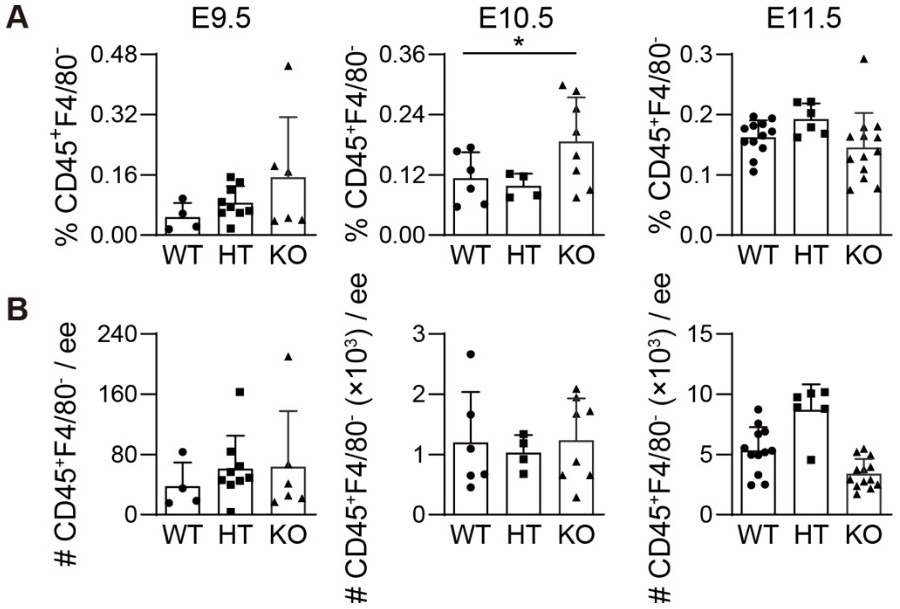

FIGURE S2 | The development of CD45$^+$F4/80$^-$ hematopoietic cells in E9.5-E11.5 Wip1$^{-/-}$ head. (A, B)The percentages and absolute numbers of CD45$^+$F480$^-$ cells in E9.5-E11.5 embryonic head. E9.5 n=4; E10.5 n=3; E11.5 n=6; **P*< 0.05.

FIGURE S3.

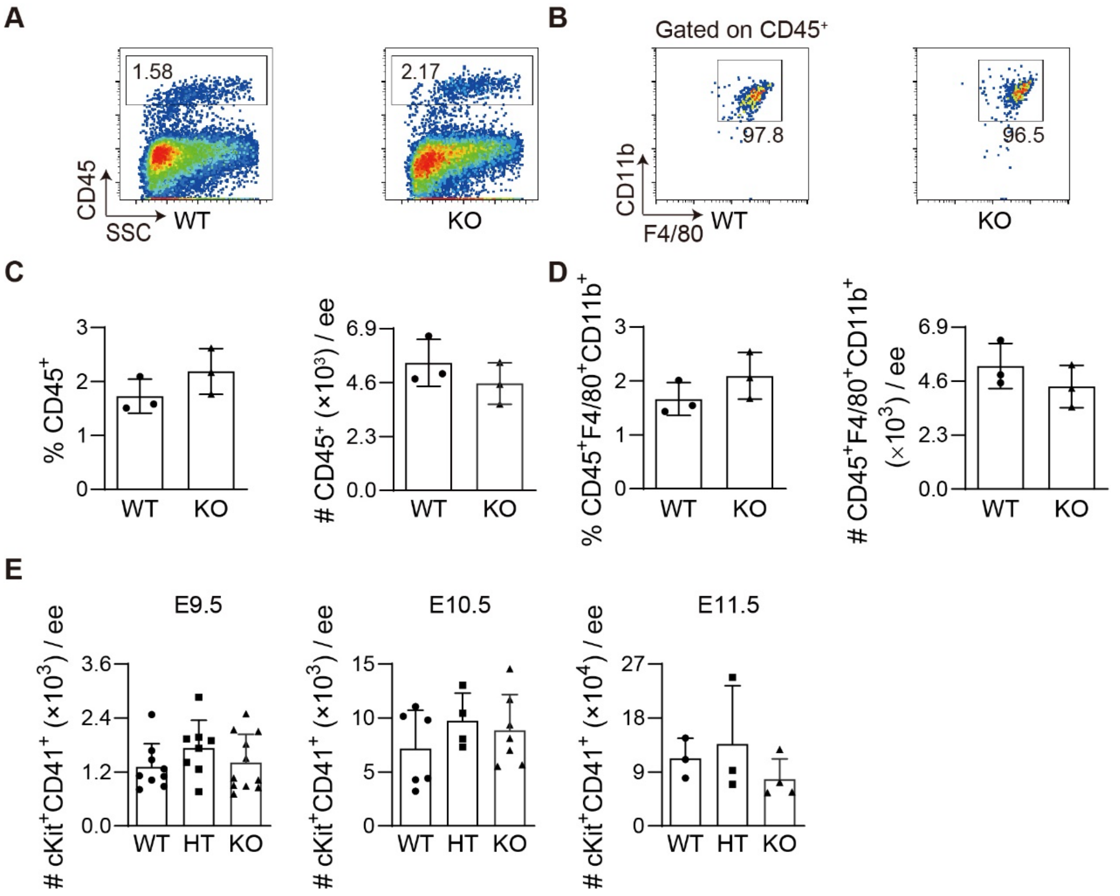

FIGURE S3 | Wip1 deletion fails to change the development of macrophages in E9.5-E11.5 YS. (**A, B**) Representative flow cytometric analysis of CD45$^+$ and CD45$^+$F480$^+$CD11b$^+$ cells in E11.5 embryonic YS. n=3. **(C, D)** The percentages and absolute numbers of CD45$^+$ cells and CD45$^+$F480$^+$CD11b$^+$ cells per head in E11.5 embryonic YS. n=3. **(E)** The absolute numbers of ckit$^+$CD41$^+$ cells in E9.5-E11.5 yolk sac. E9.5 n=5; E10.5 n=3; E11.5 n=3.

FIGURE S4.

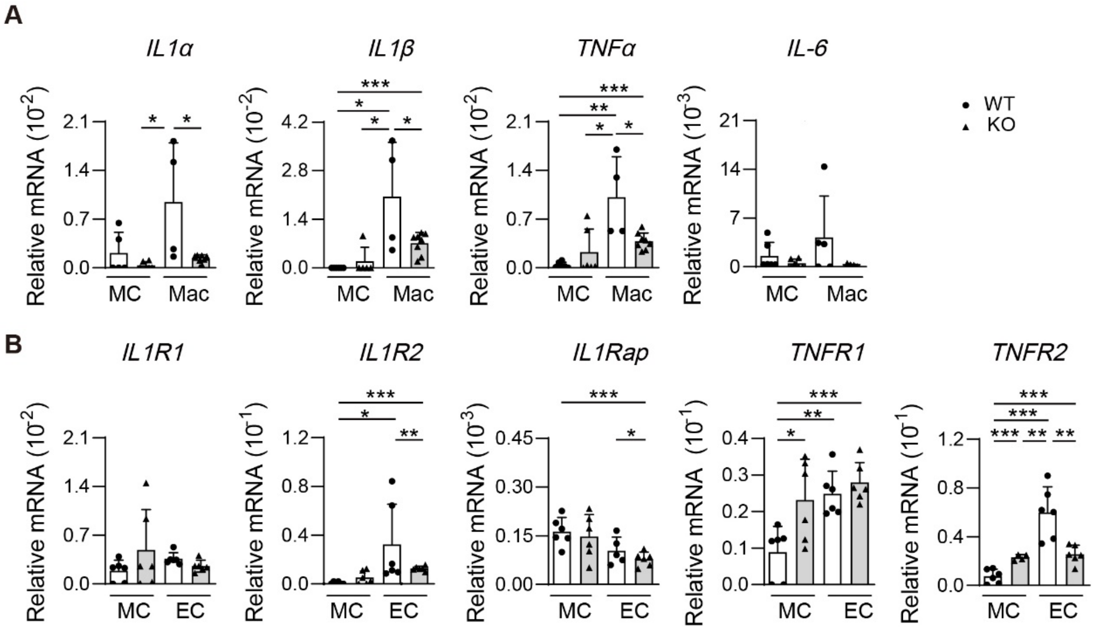

FIGURE S4 | The gene expression of pro-inflammatory pathways in the embryonic yolk sac. (A) Relative mRNA expression of *IL1α, IL1β, TNFα*, and *IL-6* normalized to actin in the macrophage (CD45$^+$F4/80$^+$CD11b$^+$, Mac) and mesenchymal cells (CD31$^-$CD45$^-$CD41$^-$, MC) of E11.5 yolk sac by qRT-PCR. n=3; **P*< 0.05, ***P*<0.01, ****P*<0.001. **(B)** Relative mRNA expression of *IL1* and *TNF* receptor genes (*IL1R1, IL1R2, IL1Rap, TNFR1, TNFR2*) in endothelial cells (CD31$^+$CD41$^-$CD45$^-$, EC) and mesenchymal cells (CD31$^-$CD45$^-$CD41$^-$, MC) of E11.5 yolk sac were determined by qRT-PCR. n=3; **P*< 0.05, ***P*<0.01, ****P*<0.001.
